# Participation of gut microbiota and bacterial translocation in chronic systemic inflammation in recently diagnosed rheumatoid arthritis patients

**DOI:** 10.1016/j.crmicr.2025.100366

**Published:** 2025-02-24

**Authors:** Catherine Dunyach-Remy, Cassandra Pouget, Yves-Marie Pers, Cécile Gaujoux-Viala, Christophe Demattei, Florian Salipante, Lucia Grenga, Jean Armengaud, Jean-Philippe Lavigne, Christian Jorgensen

**Affiliations:** a Bacterial Virulence and Chronic Infections, INSERM U1047, Univ Montpellier, Department of Microbiology and Hospital Hygiene, CHU Nîmes, Nîmes, France; b Department of Rheumatology, Stem cells, Cellular plasticity, Regenerative medicine and Immunotherapies, IRMB, INSERM UMR1183, University of Montpellier & University Hospital of Montpellier, Montpellier, France; c Desbrest Institute of Epidemiology and Public Health, University of Montpellier, INSERM, Department of Rheumatology, CHU Nîmes, Montpellier, France; d Department of Biostatistics, Epidemiology, Public Health and Innovation in Methodology (BESPIM), CHU Nîmes, Univ Montpellier, Nîmes, France; e Department of Medicines and Technologies for Health, Atomic Energy and Alternative Energies Commission (CEA), Paris-Saclay University, Bagnols-sur-Cèze, France

**Keywords:** Rheumatoid arthritis, Gut microbiota, Bacterial translocation, Intestinal permeability

## Abstract

The objective of this study was to investigate the link between gut microbiota (GM) dysbiosis, gut inflammation, and bacterial translocation (BT) in recently diagnosed rheumatoid arthritis (RA). This case-control, observational study prospectively recruited recently diagnosed (<12 months) RA patients and age-matched healthy controls (HC) from two French hospitals between July 2014 to March 2018. The primary objective was to investigate GM composition in each group using 16S rRNA sequencing and metaproteomics approaches. Three plasmatic BT markers (sCD14, LPS-binding protein, and number of 16S rRNA gene copies) and one intestinal permeability marker (I-FABP) were quantified in blood samples.

Twenty-five were included in each group, and 50 stools and blood samples were analyzed. 16S rRNA gene analysis showed an decrease in *Coprococcus* in RA patients after Body Mass Index and HLA status. Circulating bacterial DNA (number of copies of the 16S rRNA gene) and plasmatic I-FABP were higher in RA patients compared to HCs (*p* < 0.01), indicating increased BT and intestinal permeability in these patients. Metaproteomics from stool samples highlighted an increased host humoral immune response in RA, with elevated levels of inflammatory proteins (azurocidin, cathepsin G, neutrophil defensing 1). Gut inflammation may contribute to increased intestinal permeability, leading to BT into the systemic circulation and thus chronic inflammation.

## Highlights

• We show that biomarkers of bacterial translocation (circulating bacterial DNA) and intestinal permeability (I-FABP) are significantly increased in recently diagnose rheumatoid arthritis patients.
• We show decrease of *coprococcus* genus in recently diagnose rheumatoid arthritis patients with a targeted metagenomic approach.
• We highlight increase of bacterial lipid metabolism and several host inflammatory proteins in recently diagnose rheumatoid arthritis patients with a metaproteomic approach.
• We describe physiopathological mechanism (bacterial translocation from intestinal microbiota) potentially involved in systemic and secondarily local inflammation in rheumatoid arthritis patients.

## Graphical abstract

Summarized schematic illustration of the study. Among different exogenous factors (A), anti-inflammatory treatments and overweight could contribute to gut microbiota dysbiosis (B) favoring gut permeability (observed by the levels of I-FABP) (C), increased bacterial lipid metabolism (D) and local gut inflammation (E) . This early dysbiosis could contribute to BT in the systemic circulation (circularing bacterial DNA) and thus chronic inflammation (F).

**Figure:**
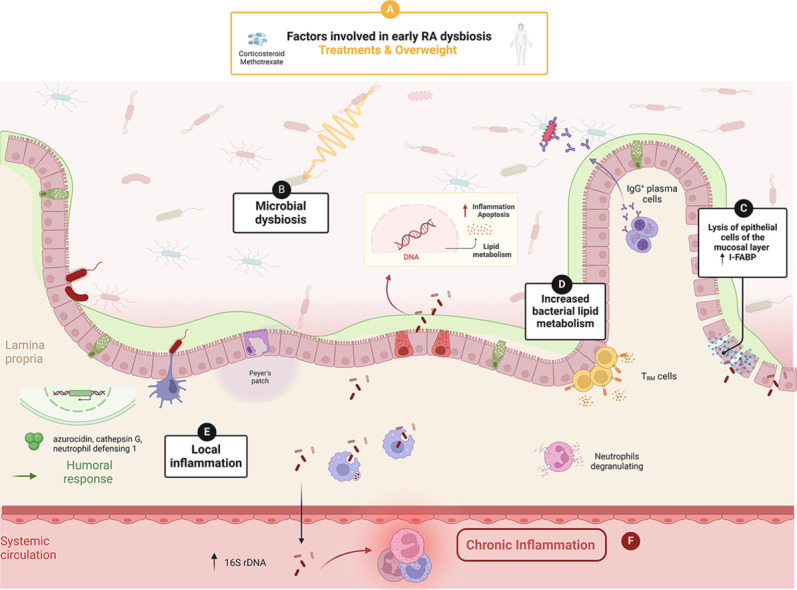

**Abbeviations**
GM: gut microbiota
RA: rheumatoid arthritis
BT: bacterial translocation
HC: healthy controls
rRNA: ribosomal RNA
LPS: Lipopolysacchariride
sCD14: soluble Cluster of Differentiation 14
I-FABP: intestinal fatty-acid binding protein (I-FABP)
BMI: Body Mass Index
HLA: Human leucocytes Antigènes
ACPA: Anti-citrullinated Protein Antibody
SFB: Segmented filamentous bacteria
DMARDs: synthetic Disease-modifying antirheumatic drugs
MTX: methotrexate
CRP: C-reactive protein
DAS28: Disease Activity Score
OTU: Operational Taxonomic Unit
GO: gene ontology
KO: KEGG ontology
FDR correction: False Discovery Rate

## 1 Introduction

Rheumatoid arthritis (RA) is a chronic inflammatory disease characterized by synovitis, leading to joints damage, systemic inflammation, and the production of autoantibodies (Guo et al., 2018). RA results from a loss of immune homeostasis, triggering an autoimmune response that includes the emergence of Anti-citullinated Protein Antibody (ACPA) (Guo et al., 2018). While the specific causes of RA remain unknown, multiple genetic and environmental factors have been associated with an increased risk of developing the disease (Yarwood et al., 2016). Notably, genetic factors such as HLA-DR4 alleles and smoking are known to elevate the risk of RA (Yarwood et al., 2016).

In vivo animal models and clinical studies have suggested that microorganisms also contribute to the etiopathogenesis of RA. Bacteria such as Porphyromonas gingivalis (Martinez-Martinez et al., 2009) and *Proteus mirabilis* (Rashid et al., 2007) have been identified in synovial fluids or membranes from RA patients. Additionally, bacterial fatty acids, cell wall proteins, peptidoglycan, muramic acid, and the QKRAA motif of the dnaJ class of heat-shock proteins from *Escherichia coli* have been observed in RA samples (van der Heijden et al., 2000). This raises the question of how bacteria or bacterial components could migrate to synovial tissue and local sites of inflammation. An emerging hypothesis to explain this possibility is bacterial translocation (BT). BT is the process by which viable and/or bacterial components cross the gastrointestinal barrier to enter the systemic circulation (Balzan et al., 2007). The principal mechanisms promoting BT include increased permeability of the intestinal mucosal barrier, deficiencies in host immune defenses, and an imbalance in the composition of gut microbiota (GM), known as dysbiosis (Balzan et al., 2007).

The GM comprises the entire complex microbial community within the human intestinal tract. It offers benefits to the host, such as strengthening gut integrity or shaping the intestinal epithelium, in addition to its evident role in nutrition. However, GM dysbiosis has been observed in RA patients, including an imbalance in Bacteroides fragilis and Clostridium sp. observed in human stools (Jeong et al., 2019). Segmented filamentous bacteria (SFB) have been highlighted as playing a major role in pathogenesis in a mouse model (Wu et al., 2010). In this model, Hsin-Jung et al. demonstrated that colonization by these bacteria in the digestive tract predisposes to the development of arthritis (Wu et al., 2010). Although poorly described, SFB in humans is closely related to the *Candidatus Savagella* genus in terms of phylogeny (Jonsson et al., 2020). Phylogenomic analysis clusters this genome with SFB genomes from mice, rats, and turkeys, but this genome is genetically distinct. Thus, GM dysbiosis could be considered a possible site of origin for the autoimmune and inflammatory processes in this pathology.

Evidence suggests that BT of endogenous bacteria from the GM could represent an important early step in the pathogenesis of chronic inflammation, as already described in other chronic pathologies such as Crohn's disease or cancers (Belkaid et al., 2017). The aim of our study was to investigate the modification of GM composition and the participation of BT in the pathology and chronicity of inflammation in the early stage of RA, prior to the use of biological, biosimilar, and targeted synthetic Disease-modifying antirheumatic drugs (DMARDs).

## 2 METHODS

### 2.1 Study population

This prospective, bicentric, case-control, observational study was conducted from July 2014 to March 2018 in the Departments of Rheumatology at Montpellier and Nîmes University Hospitals (France). Our work adhered to the guidelines of good clinical practice and the Declaration of Helsinki as revised in 2008. Written informed consent was obtained from all patients and healthy controls (HC) prior to participation. The study was approved by the ethical committee (CPP (comité de protection des personnes) Sud Méditerranée III, France, N° 2014.12.03) and registered on clinicaltrials.gov (NCT01961310).

Patients were consecutively included based on the following criteria: age ≥ 18 years old and a diagnosis of RA within the previous 12 months to ensure the inclusion of recent RA cases. Exclusion criteria included a history of digestive disease, current or recent (last 30 days) antibiotic therapy, and pregnancy. Patients were not treated with biological (b), biosimilar (bs), or targeted synthetic (ts) Disease Modifying Anti-Rheumatic Drug (bDMARDs) at the time of the study but could be treated with corticosteroids and methotrexate (Methotrexate). Corticosteroid doses ranged from 2 to 30 mg/day, and MTX treatment was limited to 15 mg weekly. HCs were volunteers without signs of autoimmune or auto-inflammatory diseases, recruited during hospital consultations. They were age-matched (± 5 years) to RA patients.

Epidemiological, clinical, and biological data were collected for all participants. The following information were recorded: age, sex, weight, height, body mass index (BMI), dietary habits (gluten-free, dairy-free, probiotics), rheumatoid factor, pain measured on a visual analog scale (VAS), ACPA, C-reactive protein (CRP), sedimentation rate, and treatments administered at the time of inclusion. The DAS28 (Disease Activity Score)-CRP score was also calculated to evaluate the RA disease activity. HLA-DR status was established. One blood sample and one stool sample were collected on the same day.

### 2.2 Sample preparation

Blood was collected in EDTA tubes to recover a total of 1 mL of plasma (centrifugation step: 1200*g* ; 10 min), which was stored at −80 °C in three different aliquots. Total DNA was extracted from 200 μL of plasma using the EZ1 DNA Tissue kit® (Qiagen, Courtaboeuf, France) according to the manufacturer's instructions. Total DNA was eluted in a final colume of 100 μL and stored at −20 °C until use. An extraction control with ultrapure molecular biology grade water was performed. Stool samples were collected directly at the hospital during the patient's inclusion visit (RA patients and healthy controls) and immediately stored at −80 °C. After thawing and manual mixing of stools, total DNA was extracted from 500 mg of stool using the QIAMP PowerFecal® kit (Qiagen) according to the manufacturer's instructions. An extraction control with ultrapure molecular biology grade water was also performed. Total DNA was eluted in a final volume of 100 μL and the concentrations of extracted DNA were checked using a QUBIT® fluorometer (Thermo Fisher Scientific, Waltham, MA, USA). Extractions were stored at −20 °C until use.

### 2.3 16S rRNA gene sequencing from gut microbiota

The bacterial communities of DNA samples extracted from stool were analyzed using a metabarcoding approach developed, optimized, and standardized by GenoScreen®. Amplicon libraries were prepared according to the Metabiote® solution v2.0, limiting amplification bias between samples and including a positive control (an artificial bacterial community called "ABCv2” componed of 15 bacterial strains and two *Archae*) and a negative control corresponding to the background noise (BF) of the total library preparation, called BF-Lib, including BF of extraction and PCR from ultrapure molecular biology grade water. Libraries were generated targeting the V3-V4 region of the 16S rRNA gene. The sequencing of the amplicon libraries was performed on a single run of Illumina MiSeq (Illumina, San Diego, USA) "paired-end" in 2 × 250 base chemistry. After validation of qualitative and quantitative parameters of the run (Cluster density, Cluster “Passing Filter”, Read, Read “Passing Filter”, Phred score, % >Q30), demultiplexing was performed by CASAVA software (Illumina) using the PERL script ConfigureBclToFactq.pl. The "merging" step or assembly of the "paired-end" reads was carried out using the FLASH tool (Magoc et al., 2011) with the following parameters: i) PCR primers search and removal (100 % nucleic identity search), ii) trimming of low-quality reads (< Q30 score corresponding to Phred score (Q)), iii) overlap area of 30 bases for assembly, iv) a 97 % nucleotide identity assembly over the entire overlap area. Similar sequences were clustered at a defined identity threshold (97 % identity for genus affiliation on the targeted 16S rRNA gene region) with Uclust v1.2.22q (Prasad et al., 2015). The identical reads were classified according to the Greengenes database (McDonald et al., 2012). An Operational Taxonomic Unit (OTU) table was generated, and each assigned OTU was expressed. To make the samples comparable, a transformation step was performed, and absolute abundances were transformed into relative abundances (%: number of reads of each OTU/total of reads). Alpha-diversity was calculated by “OTU numbers” and Shannon index, representing the taxonomic diversity of samples as the number of sequences obtained increased. Beta diversity was addressed with Bray-Curtis dissimilarity and PCoA test.

### 2.4 16S rRNA gene quantification from plasma by real-time pcr

The number of 16S rRNA gene copies was measured in plasma by qPCR following the protocol previously published (Kramski et al., 2011). Plasma was selected for the quantification of 16S rRNA gene copies to specifically target circulating cell-free bacterial DNA, which is considered a key marker of bacterial translocation. This method is the standard approach commonly used in studies investigating bacterial translocation and circulating microbial DNA. While whole blood or buffy coat could offer additional insights, particularly for detecting intracellular bacterial DNA in immune cells, our focus was on the measurement of cell-free DNA in the plasma fraction as an indicator of systemic microbial presence. It is also important to note that whole blood contains PCR inhibitors, such as hemoglobin, which can compromise the accuracy and sensitivity of bacterial DNA quantification. The amplified region was 199 bp in size. The quantification of bacterial 16S rRNA gene copies was performed using specific primers and a probe: Forward primer (16S F): 5′-AACAGGATTAGATACCCTGGTAG-3′ (nucleotide positions 780–802); Reverse primer (16S R): 5′-GGTTCTKCGCGTTGCWTC-3′ (positions 962–979, where W = *A*/T and K = G/T); and Probe: 5′-FAM-AAC7AC5TGCTCCACCGCT-BHQ1–3′ (positions 948–937). The qPCR cycling conditions were as follows: an initial denaturation at 95 °C for 10 mins, followed by 40 cycles of denaturation at 95 °C for 15 ss and annealing/extension at 60 °C for 1 min. 16S rRNA gene real-time PCR was performed twice in two biological duplicates for each sample. A negative control with molecular biology grade pure water was used. A standard curve was created from serial dilutions of plasmid DNA containing known copy numbers of the template. Two points of the standard curve were analyzed in each test to validate the PCR efficiency. The qPCR efficiency was 1.901, with an error estimated at 1.43 %. The detection limit of all runs was 10 copies per reaction. The assay precision for serial dilutions of plasmid used for the standard curve was highly reproducible, with a CV of <3.3 % for all dilutions (Kramski et al., 2011). The assays were performed using a LightCycler 480 II (Roche, Meylan, France). Absolute quantification analysis was performed with the Lightcycler 480 software (Roche), version 1.5, according to the manufacturer's recommendations.

### 2.5 Gut inflammation and permeability markers

LPS binding protein (LBP) and soluble cluster of differentiation 14 (sCD14) markers were used as biomarkers of gut inflammation. Intestinal fatty acid binding protein (I-FABP) was measured to study intestinal permeability. LBP plasma levels were measured using the Enzyme Immunoassay for Quantification of free human LBP (Enzo Life Sciences, Farmingdale, USA) ELISA kit, according to the manufacturer's recommendations. The dilution factor used was 1:800, with a detection limit of 3.125 ng/mL. Plasma levels of sCD14 were measured using the Enzyme Immunoassay for Quantification of soluble human CD14 (Enzo Life Sciences) ELISA kit, according to the manufacturer's recommendations. The dilution factor used was 1:200, with a detection limit of 1.5 ng/mL. I-FABP plasma levels were measured using an ELISA kit (Bio-Techne, Minneapolis, USA) according to the manufacturer's recommendations. The dilution factor was 1:2, with a detection limit of 47 pg/mL. Ultrapure water negative control and reference serum were used to validate each test. Quantification was achieved using a standard curve, which was generated by plotting absorbance values against known concentrations of standards. The concentration of the human marker in samples with unknown concentrations was then interpolated from this standard curve, allowing for accurate determination of the analyte concentration.

### 2.6 Metaproteomic analysis of gut microbiota

In an ancillary analysis, 12 stool samples of each group (12 HC and 12 RA) were selected based on the sufficient quantity of biological material available (over 50 mg of stool) at the end of DNA extraction for 16S rRNA gene sequencing. These samples were analyzed by metaproteomics. Proteins were extracted from 50 mg of stool samples and peptides were generated as previously described (Grenga et al., 2022). The resulting peptide pools were acidified with 0.5 % trifluoroacetic acid final concentration prior to nanoLC-MS/MS analysis with an ESI‐Q Exactive HF mass spectrometer (Thermo Fisher Scientific) coupled to an Ultimate 3000 NanoLC System (Thermo Fisher Scientific). A volume of 10 μL of peptides was injected onto a reverse phase Acclaim PepMap 100 C18 column (3 μm, 100 Å, 75 μm id × 500 mm) and resolved at a flow rate of 0.2 μL/min with a 120 min gradient of CH_3_CN (4 % to 40 %) in the presence of 0.1 % formic acid. The tandem mass spectrometer was operated with a top‐20 strategy in data‐dependent acquisition mode. Peptide molecular ions with double or triple positive charges were selected for fragmentation with a dynamic exclusion of 10 seconds as previously described (Klein et al., 2016). MS/MS spectra were interpreted using the MASCOT Daemon 2.6.0 software (Matrix Science, used with default parameters) against the NCBI non-redundant database (containing 108,307,546 sequences; downloaded from ftp://ftp.ncbi.nlm.nih.gov/blast/db/FASTA/nr.gz) following the previously described approach (Pible et al., 2020). Peptide matches with a MASCOT peptide score below a p-value of 0.05 were considered. The taxonomic composition of each fecal microbiota was evaluated based on taxon-specific peptides and taxon-to-spectrum matches (Pible et al., 2020). Peptide matches with a MASCOT peptide score below a p-value of 0.05 were considered. Proteins were validated when at least two different peptides were detected and quantified based on their spectral counts. The normalized spectral abundance factor (NSAF) was calculated by dividing the spectral count for each observed protein by the polypeptide theoretical mass in kDa. KEGG ortholog (KO) annotation of microbial protein sequences was conducted with the GhostKOALA web application (https://www.kegg.jp/ghostkoala/). Human functionally grouped gene ontology (GO terms) and pathway annotation networks were deciphered using the Cytoscape plug-in ClueGO (v2.5.4) (Bindea et al., 2009).

### 2.7 Statistical analysis

An initial analysis described the population by group (RA patients and HC). Statistical results are presented as means and standard deviations for quantitative variables with a Gaussian distribution, and medians and interquartile ranges (Q1, Q3) for other variables. For qualitative variables, the numbers and associated percentages are presented. Baseline patient characteristics were compared using a Student's *t*-test or Mann-Whitney Wilcoxon test for quantitative variables, and by a Chi-square test or Fisher's exact test for qualitative variables. Spearman's coefficients were used for correlations. All *p*-values obtained from the tests were associated with their *q*-values (*p*-values with False Discovery Rate (FDR) correction). The translocation, inflammation, and permeability markers, as well as GM analysis, were compared between the two groups using univariate analysis and a general linear model, adjusted for BMI and HLA-DR4 status, which have been described to affect both BT and gut permeability. Statistically significant differences in biological processes and GO terms identified by metaproteomics analysis were assessed using the Mann-Whitney test. The impact of steroids and MTX, alone or in combination, on BT was investigated between groups in an axillary analysis. The statistical analysis was conducted under R 3.5.1 software; R Foundation for Statistical Computing, Vienna, Austria). A statistically significant difference was considered for *p*< 0.05 for all tests.

## 3 Results

### 3.1 Patient characteristics

Between July 17, 2014 and March 29, 2018, 55 participants were initially included in the study. However, two patients were excluded due to not meeting the inclusion criteria, and three HCs were excluded as they could not be matched. Consequently, 25 recently diagnosed RA patients and 25 HCs were retained and analyzed. Table 1 (and Supplementary Table S1) presents the demographics and clinical characteristics of the 50 participants. The only statistical significant difference between the two groups was BMI, which was significantly higher in RA patients compared to HCs (27.4 ± 6.3 vs 24.2 ± 3.2 respectively; *p* = 0.029). In contrast, the presence of HLA-DR4 alleles did not significantly differ between the groups (33 % vs 29 %, respectively).

**Table 1:** Demographic and clinical characteristics of the study population (*n* = 50).

	RA patients		Healthy Controls		
	N	n (%) or mean (sd) or median [Q1-Q3]	N	n (%) or mean (sd) or median [Q1-Q3]	*p*
Female sex	25	14 (56)	25	13 (52)	1
Age, years mean	25	57.0 ± 13.0	25	57.4 ± 12.8	0.913
Body mass index, kg/m^2^	25	27.4 ± 6.3	25	24.2 ± 3.2	0.029
HLA-DRB1 alleles	24	8 (32)	24	7 (28)	1
Time since first diagnosis, (month)	25	2.30 [0.25–5.42]	–	–	–
Rheumatoid factor	25	14 (56)	–	–	–
Positive Anti-Citrullinated Protein Antibody (ACPA)	25	15 (60)	–	–	–
CRP > norm	25	19 (76)	–	–	
CRP values (mg/L)	25	24.4 [10–52]	–	–	–
Patient activity assessment (VAS) (cm)	21	6.5 [3–8]	–	–	–
Patient condition (VAS) (cm)	21	5 [3–7]	–	–	–
Number of painful joints	23	4 [3–11]	–	–	–
Number of swollen joints	23	2 [0–5]	–	–	–
DAS28 score	15	4.92 [4.18–5.61]	–	–	–
DAS28 score interpretation Dietary particularities	15	High(*n* = 5) Medium(*n* = 9) Remission (*n* = 1)	–		
Gluten free		0 (0)		1 (4)	NA
Diary-free		0 (0)		0 (0)	1
Probiotics		0 (0)		1 (0)	NA
Treatment	24		–		
Corticosteroid only		9 (37.5)		–	
methotrexate (Methotrexate) only		3 (12.5)		–	
Both		7 (29.2)		–	
cs DMARDs			10 (41.7)		–
None			5 (20.8)		–

Data are presented as mean ±SD, number (%) or median [IQR]. *P* values were calculated using Student test or Mann-Whitney Wilcoxon test for quantitative variables, and Chi-2 test or Fisher for qualitative variables. NA, non-available; CRP, C-reactive protein; VAS, Visual analog scale; DAS28-CRP (Desease Activity Score); conventional synthetic DMARDs (Disease Modifying Anti-Rheumatic Drug).

### 3.2 Relative abondance of some bacterial genera in gut microbiota is different in ra patients

A total of 1976,231 reads were obtained, corresponding to an average of 38,000 ± 2026 reads per sample. In total, 5300 OTUs were identified in the fecal samples, of which 80 % were assigned to the genus level. The ABCv2 control presented 45,495 reads with 97 % identity after assembling, and relative abondances compatible with high-quality sequencing (Supplementary Fig. 1a). Shannon's diversity index, abundance and equal distribution of the OTU, did not show significant differences between the RA group and HCs (4.9 [4.2–5.2] vs 4.8 [4.4–5.5], respectively, *p* = 0.461) (Supplementary Fig. 1b). Similarly, the β-diversity did not demonstrate any clusters to distinguish RA patients from HC (*p* = 0.11) (Supplementary Fig. 1c). In the negative control, 99 reads were obtained with 97 % identity after assembling, showing minimal contamination. Bacterial abundances are reported as percentages of the total microbiota composition within each sample.

**Fig. 1:**
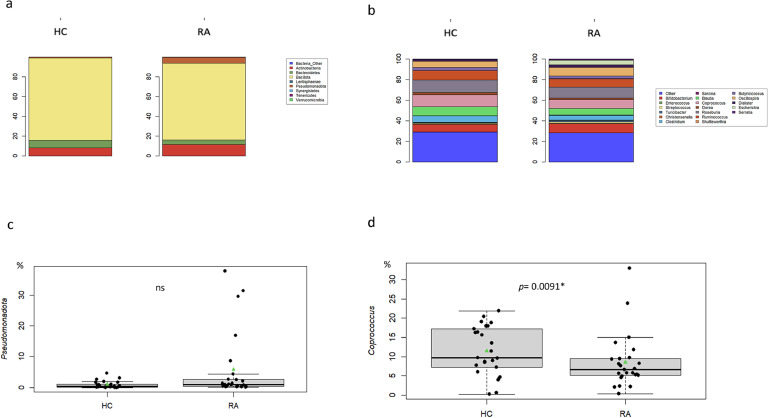
Description of bacterial communities of gut microbiota in rheumatoid arthritis (RA) patients (RA) (*n* = 25) and Healthy Controls (HC) (*n* = 25). (a) Barplot of overall repartition of phyla in gut microbiota for RA and HC. (b) Barplot of average relative frequencies of genera. Only genus > 1 % of OTUs in at least one stool from RA patients and HC were represented. The remaining genus are added to the group “other”. (c) Boxplot of Pseudomonadota phylum abundance (d) Boxplot of *Coprococcus* genus abundance*.* Significance presented in the graphs was calculated by univariate analysis and by a general linear model, adjusted on the BMI and HLA DR4 status. ns; non-significant; *, *p* = 0.0091.

The overall gut bacterial community was mainly composed of eight phyla and 60 families. The profiles of intestinal bacterial communities for each group at the phylum level are presented in Fig. 1a and Supplementary Fig. 2. In both groups, the dominant phyla were Bacillota (Firmicutes) (82.3 (71.2, 89.3); 83.8 (77.7, 95.9) for RA and HC, respectively), Actinomycetota (Actinobacteria) (5.3 (1.91, 17.5); 4.68 (1.05, 14.8) for RA and HC, respectively)*,* Pseudomonadota (Proteobacteria) (1.01 (0.47, 2.3); 0.41 (0.14, 1.07) for RA and HC, respectively) and Bacteroidota (Bacteroidetes) (2,34 (0.44, 5.32); 1.61 (0.1, 8.1) for RA and HC, respectively). Results revealed that the relative abundance of Bacillota (Firmicutes) showed a non-significant trend of being slightly higher in HCs than in RA patients (*p* = 0.16). In contrast, Pseudomonadota (Proteobacteria) was significantly more abundant in RA patients (*p* = 0.03). Percentages of Actinomycetota (Actinobacteria) and Bacteroidota (Bacteroidetes) were not significantly different between the two groups (*p* = 0.39 and *p* = 0.72, respectively). After adjusting for BMI, the proportion of Pseudomonadota (Proteobacteria) remained significantly higher in RA patients (*p* = 0.0195). However, after adjusting for both BMI and HLA status, no difference was observed (Fig. 1a-c) (Supplementary Fig. 2).

A total of 94 genus were identified. The profiles of intestinal bacterial communities for each group at the genus level are represented in Fig. 1b (Supplementary Fig. 3). Results highlighted a significant different in the relative abundance of *Actinomyces* (*p* = 0.006; *q* = 0.29), *Butyrivibrio* (*p* = 0.02; *q* = 0.53), *Coprococcus* (*p* = 0.049; *q* = 0.56), *Peptococcus* (*p* = 0.02; *q* = 0.53), *Campylobacter* (*p* = 0.04; *q* = 0.56) and *Escherichia* (*p*= 0,0,000,666; *q* = 0,006) in RA patients compared to HCs. After adjusting for BMI, *Actinomyces, Coprococcus* and *Escherichia* remained significantly different in RA patients (*p* = 0.0365; *p* = 0.0492; *p* = 0.00658, respectively). *Coprococcus* was still significantly decrease in RA patients after adjusting for both BMI and HLA (*p*= 0.0091) (Fig. 1b,d). By focusing on the differential impact of various treatment strategies on bacterial genera within the microbiota, the analysis reveals some shifts in GM composition. Notably, the relative abundance of *Clostridium* was significantly elevated in untreated patients and in patients treated with methotrexate (Methotrexate) alone (*p*= 0.007) (Table 2). Similarly, *Oscillospira* showed a significant increase (*p*= 0.048), with the untreated (11.9 %) and methotrexate (Methotrexate)-only groups (9.4 %) displaying higher levels relative to the corticosteroid group. In contrast, there were no statistically significant differences in the relative abundance of *Coprococcus* across the treatment groups (*p*> 0.05), suggesting that this genus may be similarly affected by the various treatments described in this study (Table 2).

**Table 2:** Impact of corticosteroids and methotrexate (Methotrexate) treatment on gut microbiota.

**Characteristic**	**Both**, N = 7^1^	**Corticosteroid only**, N = 9^1^	**methotrexate (Methotrexate) only**, N = 3^1^	**None**, N = 5^1^	**p-value**^2^
*Peptococcus (%)*	0.00 (0.00, 2.16)	0.00 (0.00, 0.00)	0.00 (0.00, 0.95)	0.00 (0.00, 0.01)	0.2
*Bifidobacterium (%)*	8 (5, 18)	5 (2, 16)	5 (3, 6)	1 (1, 1)	0.059
*Clostridium (%)*	3.6 (2.8, 4.9)	2.0 (1.9, 2.2)	7.5 (6.3, 9.7)	8.4 (4.4, 8.5)	0.007*
*Dehalobacterium (%)*	0.00 (0.00, 0.00)	0.00 (0.00, 0.00)	0.02 (0.01, 0.02)	0.00 (0.00, 0.02)	0.077
*Oscillospira (%)*	1.7 (1.3, 9.0)	4.1 (2.7, 5.1)	9.4 (9.0, 10.2)	11.9 (11.4, 13.3)	0.048*
*Coprococcus (%)*	9 (7, 13)	7 (6, 8)	8 (5, 16)	5 (2,5)	0,2

1 Median (IQ1; IQ3).

2 Kruskal-Wallis rank sum test.

⁎ Significant P values (<0.05).

### 3.3 Bacterial translocation and gut permeability markers are increased in RA patients

**Fig. 2:**
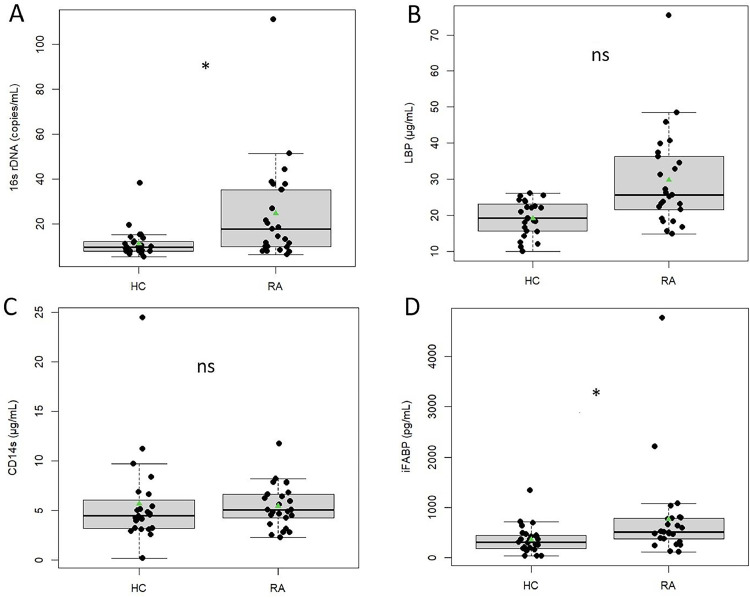
Comparison of bacterial translocation (BT) and gut permeability markers in patients with rheumatoid arthritis (RA) and healthy controls (HC). BT markers are compared between RA patients (*n* = 25) and HC (*n* = 25) for (A) 16S rDNA, (B) LPS-binding protein (LBP), (C) sCD14 and for (D) I-FABP. Significance presented in the graphs was calculated by univariate analysis and by a general linear model, adjusted on the BMI and HLA DR4 status. ns; non-significant; *, *p* ≤ 0.05.

The plasma levels of circulating bacterial DNA (16S rRNA gene copies) and gut inflammatory and permeability markers (LBP, CD14 s, I-FABP) were measured in RA patients and HCs (Table 3; Fig. 2). For all ELISA tests (LPB, CD14 s, I-FABP), the negative controls and reference sera values validated the tests. Firstly, the analysis examined potential drug-dependent effects on bacterial translocation markers across different treatment subgroups (Table 3). Regarding the 16S rRNA gene copies per µL, no significant differences were observed among the groups. Similarly, I-FABP levels did not show significant variation, with concentrations ranging from 475 pg/mL (292, 491) in the methotrexate (Methotrexate)-only group to 627 pg/mL (475, 798) in the corticosteroid-only group (*p* = 0.5) (Table 3). sCD14 concentrations also did not differ significantly, ranging from 4.25 µg/mL (3.22, 4.88) in the "MTX+corticosteroid" group to 6.40 µg/mL (4.84, 6.83) in the corticosteroid-only group (*p* = 0.2). Although LBP levels approached statistical significance (*p* = 0.073), the highest median value was noted in the corticosteroid-only group (35 µg/ml) (Table 3). Given the lack of significant differences within the treatment subgroups, we subsequently combined these groups and compared these BT biomarkers to those of HCs. Plasma levels of circulating bacteria DNA were significantly higher in RA patients compared to HCs (17.6 copies/μL [9.9–35.3] vs 9.6 [3.22–5.74], respectively; *p* = 0.007). The significant difference between the two groups remained after adjustment for BMI alone (*p* = 0.012) and for HLA status plus BMI (*p* = 0.014) (Fig. 2a). Negative controls (extraction and PCR controls) presented a copies number < 3 copies/ μL, corresponding to contamination of PCR polymerase.

**Table 3:** Impact of corticosteroids and methotrexate (Methotrexate) treatment on bacterial translocation markers.

**Characteristic**	**Both**, N = 7^1^	**Corticosteroid only**, N = 9^1^	**methotrexate (Methotrexate) only**, N = 3^1^	**None**, N = 5^1^	**p-value**^2^
16S rRNA gene (copies/µL)	35 (13, 38)	14 (10, 38)	19 (14, 23)	12 (8, 13)	0.4
I-FABP (pg/ml)	499 (311, 772)	627 (475, 798)	475 (292, 491)	583 (316, 654)	0.5
sCD14 (µg/ml)	4.25 (3.22, 4.88)	6.40 (4.84, 6.83)	4.93 (3.87, 5.46)	5.63 (4.56, 7.88)	0.2
LBP (µg/ml)	22 (17, 23)	35 (23, 41)	33 (30, 39)	26 (19, 31)	0.073

1 Median (IQ1; IQ3).

2 Kruskal-Wallis rank sum test.

LBP was also significantly higher in RA patients compared to HCs (25.6 μg/mL [21.5–36.2] vs 19.2 µg/mL [15.6–22.8], respectively; *p* = 0.01). However, when values were adjusted for BMI or both BMI and HLA status, no differences were noted (*p* = 0.30 and 0.73, respectively) (Fig. 2b). Moreover, no differences in sCD14 values between RA patients and HCs were observed, regardless of HLA status (Fig. 2c).

I-FABP levels were significantly higher in RA patients compared to HCs (507 pg/mL [371–779] vs 301 pg/mL [176–437], respectively; *p* = 0.04), even after adjustment for BMI (*p* = 0.039) or both BMI and HLA status (*p* = 0.047) (Fig. 2d).

Levels of these biomarkers was assessed in relation to DAS28 scores (High vs Moderate activity), and no significant differences between these two groups were observed (*p* > 0.05).

Finally, the correlation between these biomarkers and the abundance of the genus Coprococcus, which is significantly reduced in RA patients, was assessed. Spearman's coefficient correlation indicates that there is no correlation between 16S rRNA gene copies and Coprococcus abundance (*r*< 0.5). The same applies to iFABP (*r*< 0.5). However, Fig. 4 shows that RA patients with low Coprococcus abundance exhibit a significantly higher number of 16S rRNA gene copies compared to healthy volunteers with low *Coprococcus* abundance.

### 3.4 Bacterial lipid metabolism and several host inflammatory proteins are increased as shown by metaproteomics

**Fig. 3:**
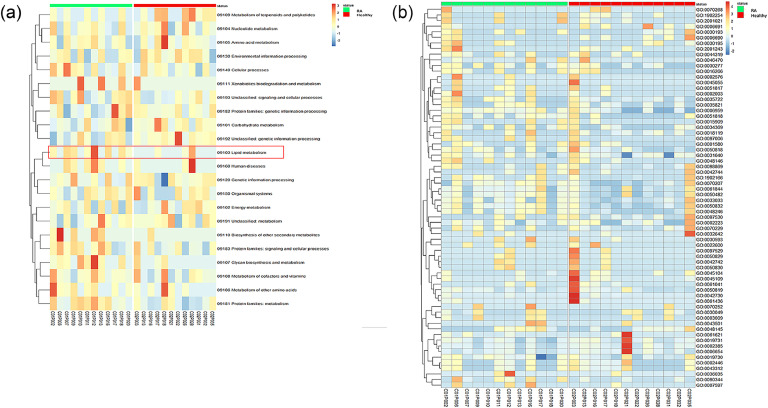
Heatmap of standardized proteins composition of fecal samples patients with rheumatoid arthritis (RA) (*n* = 12) and healthy controls (*n* = 12) stool. Proteins pathways are represented through all proteins detected (with at least 2 Spectral Count (SC)) and identified by metaproteomics analysis. Standardized relative frequencies of proteins pathways are used in order to see variations between groups even for low-abundant proteins. Proteins pathways are classified according to agglomerative hierarchical clustering with complete linkage. (A) the bacteria level or at (B) the host level.

Metaproteome analyzes enabled the simultaneous assessment of synthetized human proteins and microbial metabolic pathways. Several KEGG biological processes were identified through the integration of bacterial protein signals detected in fecal samples (Fig. 3). Processes such as lipid metabolism, metabolism of terpenoids and polyketides, biosynthesis of secondary metabolites, and xenobiotics metabolism were predominant. Only proteins involved in lipid metabolism displayed significantly increased levels in RA patients (*p* = 0.021, Mann Whitney test). The metabolism of terpenoids and polyketides showed a decreased relative abundance in RA patients (Fig. 3a). Functional profiling of human proteins present in the fecal samples of both groups is represented in Fig. 3b. In total, 137 GO terms were identified. Some of the proteins with the highest abundance identified in RA group belonged to the GO term “humoral immune response” (GO:0,006,959). This GO term is significantly more abundant in the RA group than in controls (*p* = 0.01, Mann Whitney test), corresponding to an increase in the abundance of inflammatory proteins (azurocidin, cathepsin G, neutrophil defensin 1, immunoglobulin J chain precursor and lysozyme). In the context of this particular experiment, the impact of different treatment types could not be adequately analyzed due to the insufficient sample sizes of the subgroups (corticosteroid only *n* = 4; Methotrexate only *n* = 0; none *n* = 5 and both *n* = 3).

## 4 Discussion

RA is an autoimmune disease triggered by numerous factors, including microbial ones (Bindea et al., 2009). Several studies have shown that differences in GM composition are closely linked to RA patients (Jeong et al., 2019), whereas the influence of BT elements from the GM on RA inflammation remains unclear (Martinez-Martinez et al., 2009). In this study, using a large panel of explorative tools (BT and intestinal permeability markers, 16SrRNA gene sequencing, and metaproteomic approaches), we investigated the gut microbiota and the presence of BT and its impact in RA patients compared to HCs. Surprisingly, the groups presented an equivalent presence of the HLA-DR4 allele, contrary to a previously reported study (Yarwood et al., 2016). The RA group demonstrated significant inflammation and higher BMI, confirming previous data (Bindea et al., 2009) (Table 1). Gut dysbiosis and BT were clearly present in this recently diagnosed RA population, even though overweight and HLA-DR4 status are known to increase BT and to induce GM dysbiosis (Liu et al., 2021; Andeweg et al., 2021; Asquith et al., 2019; Cuevas-Sierra et al., 2019).

**Fig. 4:**
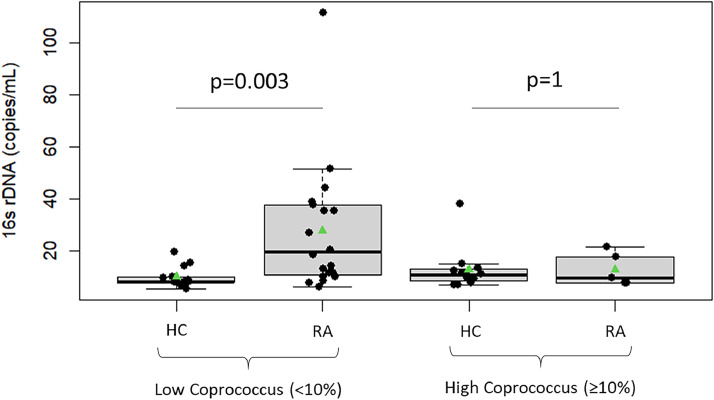
Comparison of number of copies of 16 s rRNA gene in patients with rheumatoid arthritis (RA) (*n* = 25) and healthy controls (HC) (*n* = 25) in accordance to *Coprococcus* genus abundance (Low *Coprococcus* (<10 %) and High *Coprococcus* (≥10░%)). Significance presented in the graphs was calculated by univariate analysis and by a general linear model. *p* values are indicated on graphs.

The pathophysiological mechanisms by which GM is associated with arthritis are likely multifactorial. Proposed mechanisms include activation of antigen-presenting cells through effects on TLRs or NLRs, the ability to produce citrullination of peptides by enzymatic action, antigenic mimicry, alterations in permeability of intestinal mucosal, control of the host immune system (triggering T cell differentiation), and increased T helper type 17-mediated mucosal inflammation. GM dysbiosis is a hallmark of RA, yet the distinct composition of this GM remains controversial (Jeong et al., 2019). Although SFB have been implicated in the development of arthritis in mice, the *Candidatus Savagella* genus (close to SFB) was no detected in either group. No data have been clearly established in humans, likely due to the difficulty in identifying this genus in the human intestine (Wu et al., 2010; Jonsson et al., 2020), as observed in our study. Concerning *Proteus,* this genus was also not deteted in the two studied groups (Fig. 1). This is not surprising, as no recent data have definitively confirmed the role of *Proteus* in the development of RA since the first report by Rashid et al. (2007). On the other hand, an increased abundance of *Prevotella copri* has been associated with aggravated RA prognosis (Alpizar-Rodriguez et al., 2019), this genus is not found differentially present in the RA patients. In our study, however, *Escherichia* (except after BMI and HLA status adjustment) were significantly increased in the stools of RA patients compared to those of HCs, as observed in other inflammatory diseases such as Crohn's disease and Alzheimer's disease (Ma et al., 2022; D'Argenio et al., 2022). Indeed, *Escherichia* has been observed in significantly higher abundance in collagen-induced arthritis mice (Wang et al., 2020), further linking its presence to inflammatory processes. In contrast, *Coprococcus* was significantly reduced in the stools of RA patients (Fig. 1d). *Coprococcus* has been reported as decreased in RA mouse models (Wang et al., 2022) but it has been found to be increased in RA patients (Gupta et al., 2021). *Coprococcus* species are butyrate producers, which have anti-inflammatory properties that help maintain the integrity of the intestinal epithelial barrier explaining the increase of bacterial translocation in RA patients with low abundance of *Coprococcus* in their microbiota (Fig. 4). While low concentrations of butyrate enhance cell proliferation and provide protective effects, high concentrations may induce apoptosis and exacerbate immune cell activation, contributing to an exaggerated inflammatory response (Liu et al., 2018). This dual effect of butyrate suggests that the role of *Coprococcus* in RA may depend on the balance of its metabolites.

*Bifidobacteria* spp. is one of the major commensals that impact immunity and promote T cell maturation (Jeong et al., 2021). A study has shown that Th17 cells were absent in germ-free mice and were induced by specific microbes (Wu et al., 2010). The gut microbiota mediates the Th17 response through 2,6-sialyl ligands, where 2,6-sialyltransferase deficiency induced mucosal Th17 responses. Pathological Th17 cells can also be promoted by *Actinobacterium, Eggerthella lenta,* and *Fusobacterium nucleatum* via short-chain fatty acids (SCFA) or butyrate (Wang et al., 2022).

Other studies compared fecal microbiota from patients with early RA to the microbiota of patients with non-inflammatory pain using oligonucleotide probe against the 16S rRNA gene. Patients with early RA had significantly fewer bacteria belonging to the genera *Bacteroides, Prevotella,* and *Porphyromonas* (Vaahtovuo et al., 2008). A second study reported that early RA patients were more likely to harbor *Prevotella copri* compared to healthy subjects. *P. copri* would alter intestinal permeability and lead to bacteria penetration and/or its components throughout the body; this is one of the proposed mechanisms that link dysbiosis with the pathogenesis of arthritis (Scher et al., 2013). We did not confirm the “classical” dysbiosis described in the literature with increased *P. copri* (Alpizar-Rodriguez et al., 2029) or *P. gingivalis* (Sato et al., 2017) (Fig. 1). However, most clinical studies do not consider HLA-DR4 status and BMI, which are proven causes of GM dysbiosis (Asquith et al., 2019; Cuevas-Sierra et al., 2019). Therefore, changes in GM in several pathologies, particularly in RA, are closely linked to uncontrolled and increased host inflammation (Jubair et al, 2018). We now establish a clear relationship between gut bacteria and microbial-mediated metabolites that act as signaling molecules to induce inflammation. Finally, the high relative abundance of Bacillota (Firmicutes) observed in all samples is consistent with previous reports on gut microbiota composition in inflammatory states, though the biological implications of this finding warrant further investigation.

Although gut microbial translocation and its relationship to gut microbiota dysbiosis and immune activation has been correlated in other diseases (e.g. HIV infections) (Xie et al, 2021), its association with RA and its progression are not well understood, especially for recently diagnosed patients. In this study, we measured both indirect (sCD14 and LPS-binding protein) and direct (16S rRNA gene) bacterial markers of BT. In contrast to Audo et al., who described increased plasma levels of sCD14 and LBP in patients suffering from long-term RA, this difference was not observed in our recently diagnosed patients (Audo et al, 2022) (Fig. 2). However, we showed that the 16S rRNA gene significantly increased in our RA group, suggesting that BT was clearly present in the early stage of the disease. These results were supported by high plasma levels of I-FABP in this population, as previously described (Chen et al, 2020), corroborating an intestinal epithelial cell injury leading to increased permeability (Fig. 2).

Few studies have analyzed metabolites produced by GM in RA patients (Chen et al, 2021)_._ Using a metagenome-wide association study, Kishikawa et al. found that various bacterial biological pathways related to fatty acid biosynthesis and glycosaminoglycan degradation were enriched in the RA group (Kishikawa et al, 2020). Our study confirmed that bacterial pathways, particularly lipid metabolism, displayed significantly increased levels in RA patients. This pathway includes bacterial lipolytic enzymes that hydrolyse lipids from the host cell to release free fatty acids (FFA) (Fig. 3). Lipids can modify the GM composition, as shown in overweight population, and bacteria of this GM can metabolize lipids (Ridlon et al, 2006).

Bacterial metabolites, such as SCFAs, secondary bile acids, lactic acid, and bacteriocins, have antimicrobial activities. SCFAs are produced by fermentation of indigestible carbohydrates by some commensals (like *Faecalibacterium prausnitzii, Roseburia intestinalis,* and *Anaerostipes butyraticus*) and maintain intestinal homeostasis in the normal colon by participating in intestinal repair. Moreover, acetate produced by *Bifidobacteria* spp., maintains gut–epithelial barrier function and regulates intestinal inflammation (Campbell et al, 2023).

The inflammatory consequences of this microbiota-lipid interrelation remain unexplored. In irritable bowel diseases (IBD) and obesity, FFA production has been linked to insulin resistance, inflammation, and apoptosis (Kimura et al., 2013). Here, we similarly identified host cell pathways that are modulated by RA and showed that the humoral immune response was significantly higher in fecal samples of RA patients. Proteins such as azurocidin, cathepsin G, and neutrophil defensin 1 were notably increased (Fig. 3). Azurocidin acts as a chemoattractant and activator of monocytes/macrophages. High serum levels of this protein have been described in RA (Chapman et al, 2019). Cathepsin G is a serine protease that controls the functional state of immune cells. It is involved in RA and inflammatory processes via matrix degradation. This protein is synthesized by macrophages, osteoclasts, synovial fibroblasts, and chondrocytes, which are involved in joint inflammation and cartilage destruction (Behl et al, 2022). High blood levels of cathepsin G in RA patient are well known (Skoumal et al, 2005), but this is the first description of the production of this protein in RA fecal samples (Fig. 3). Finally, many studies have demonstrated that neutrophil defensin plays a critical role in the pathophysiology of RA. Neutrophil-derived α-defensin-1 has the potential to modulate inflammatory, degradative, and invasive behavior in RA (Ahn et al, 2013). The presence of these proteins in stools confirmed a high inflammatory status, even under treatment.

Anti-rheumatic drugs are key to reducing joint inflammation, pain and preventing joint destruction, and these drugs show a reciprocal connection with inflammation and GM, influencing therapeutic outcomes (Weersma et al, 2020). Indeed, these drugs are involved in GM alteration (Nayak et al, 2021). Our study assessed the impact of two commonly used therapies (MTX and corticoids, only and in combination) on GM. Corticosteroid treatment was associated with a lower gut abundance of *Clostridium* and *Oscillospira* (Table 2)*. Oscillospira* has a negative association with BMI. Their decrease in our population is certainly related to the overweight status of the RA patients (Yang et al, 2021).

Some study limitations should be highlighted, such as the constraints placed on the low number of patients involved in the research project, the high percentage of people in the RA group with n abnormal BMI, and the very specific profile of included patients (early RA), which not allow for comparison with published data that often focus on RA patients with a later diagnosis.

## 5 Conclusion

This study provides compelling evidence that intestinal bacteria play a significant role in the pathophysiology of RA through their involvement in lipid metabolism pathways. These pathways not only amplify the host's inflammatory response by promoting the production of pro-inflammatory proteins but also increase intestinal permeability, and facilitate BT. Our findings suggest that BT is a critical mechanism in the perpetuation of chronic systemic inflammation in RA. By highlighting the connection between gut microbial activity and RA pathology, this study offers valuable insights into how gut-derived factors may influence disease progression. The elucidation of these mechanisms underscores the potential of targeting intestinal microbiota and their metabolic products as a novel therapeutic strategy for managing RA. Specifically, the importance of lipid metabolism pathways and BT emerges as key areas for future intervention.

## Funding

This research was funded by the GCS Montpellier-Nîmes, grant AOI number (2014-A01753–44). The funder had no role in study design, data collection and analysis, decision to publish or preparation of the manuscript.

## Ethics approval and consent to participate

The study was conducted in accordance with the Declaration of Helsinki, and approved by the Institutional Review Board. The study was approved by the ethical committee (CPP Sud Méditerranée III, France, N° 2014.12.03) and registered on clinicaltrials.gov (NCT01961310). Written informed consent was obtained from each participant.

### Data references

DNA sequences are accessible in Bioproject PRJNA1006174, BioSampe SAMN37015268; SRA accession numbers SRX21430554 to SRX21430603. The mass spectrometry proteomics data have been deposited to the ProteomeXchange Consortium via the PRIDE partner repository with the dataset identifier PXD045023 and 10.6019/PXD045023. The link is https://www.ebi.ac.uk/pride/archive. This dataset is accessible for the reviewers with the username reviewer_pxd045023@ebi.ac.uk and password **tSRh9Ayw**.

## Declaration of competing interest

The authors declare no competing interests.

## Data Availability

Data will be made available on request.

## Appendix Supplementary materials

Image, application 1

Image, application 2

Image, application 3

Image, application 4

Image, application 5
