# Supplementary material for: Participation of gut microbiota and bacterial translocation in chronic systemic inflammation in recently diagnosed rheumatoid arthritis patients
Source: Curr Res Microb Sci. 2025 Feb 24;8:100366. doi: 10.1016/j.crmicr.2025.100366 (PMC11928969; doi:10.1016/j.crmicr.2025.100366)
Supplement: Supplementary file 1 [file mmc1.docx]

**Supplementary figure 2:** Stacked bar plot representing relative abundance of phyla in gut microbiota in rheumatoid arthritis (RA) patients (RA) (n=25) and Healthy Controls (HC) (n=25).

**Supplementary figure 3:** Stacked bar plot representing relative abundance of genus in gut microbiota in rheumatoid arthritis (RA) patients (RA) (n=25) and Healthy Controls (HC) (n=25). Only genus > 1% of OTUs from RA patients and HC were represented. The remaining genus are added to the group “other”.
