# Supplementary material for: Participation of gut microbiota and bacterial translocation in chronic systemic inflammation in recently diagnosed rheumatoid arthritis patients
Source: Curr Res Microb Sci. 2025 Feb 24;8:100366. doi: 10.1016/j.crmicr.2025.100366 (PMC11928969; doi:10.1016/j.crmicr.2025.100366)
Supplement: Supplementary file 2 [file mmc2.docx]

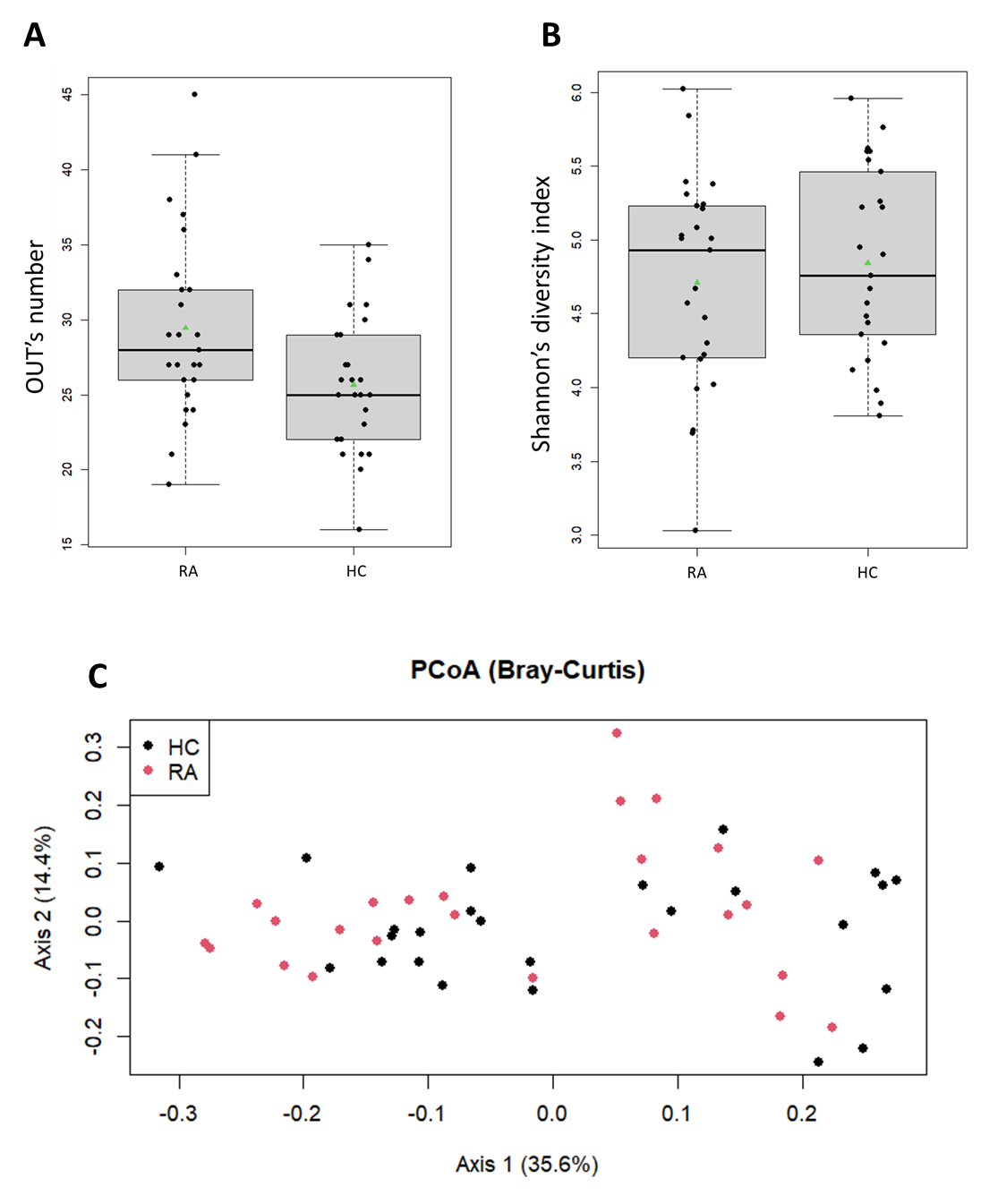


**Figure S1:** Comparison of general characteristics of metabarcoding sequencing based on stool samples of RA patients and HC. OTUs number (A), Shannon’s diversity index (B) and β-diversity by PCoA test (C) were compared between RA patients (n = 25) and HC (n = 25). Representation in dox plots - Mann-Whitney Wilcoxon test were used.
