# Supplementary figures and images for: Participation of gut microbiota and bacterial translocation in chronic systemic inflammation in recently diagnosed rheumatoid arthritis patients
Source: Curr Res Microb Sci. 2025 Feb 24;8:100366. doi: 10.1016/j.crmicr.2025.100366 (PMC11928969; doi:10.1016/j.crmicr.2025.100366)

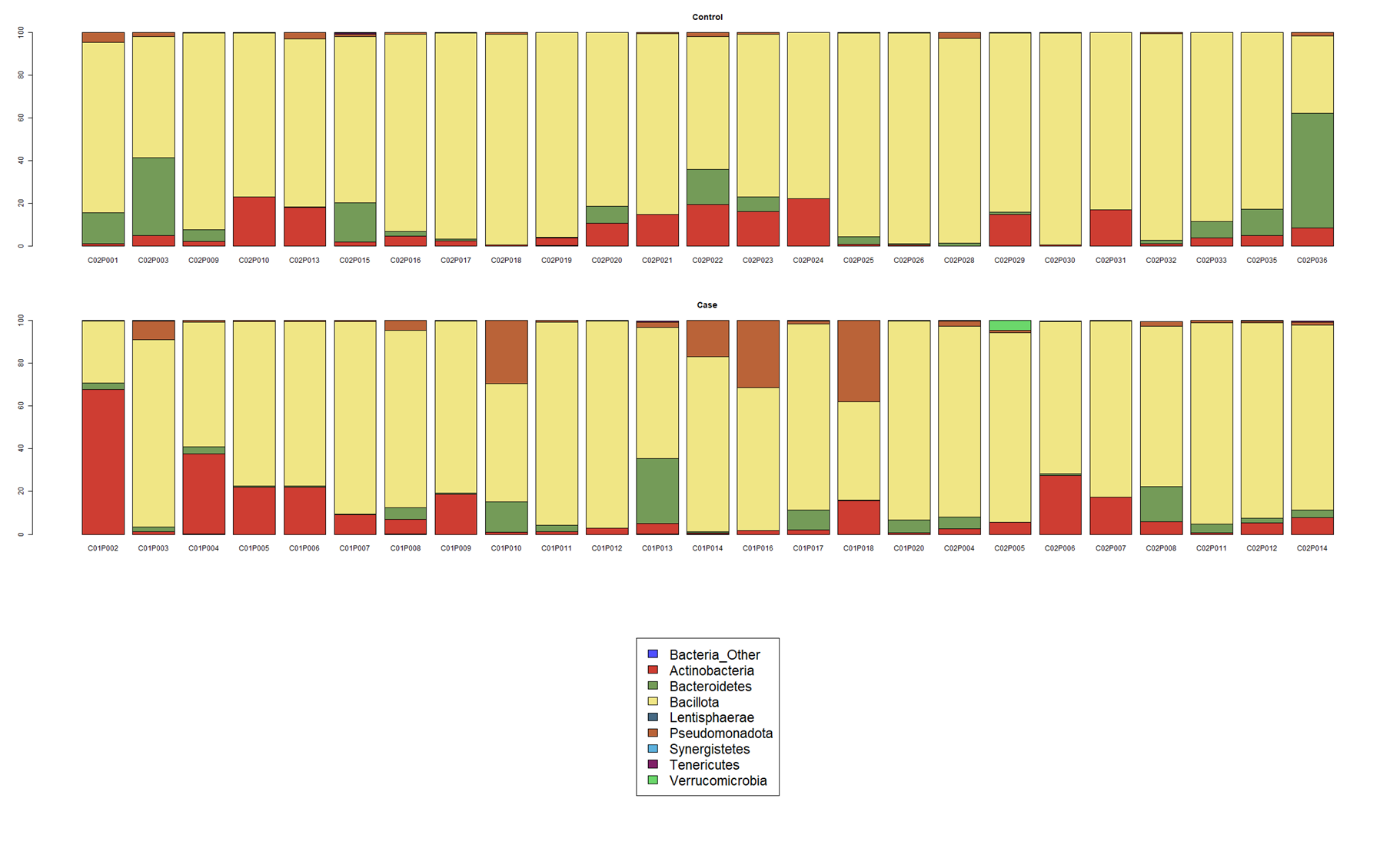

Supplement: Supplementary file 3 [file mmc3.zip › mmc3.png]

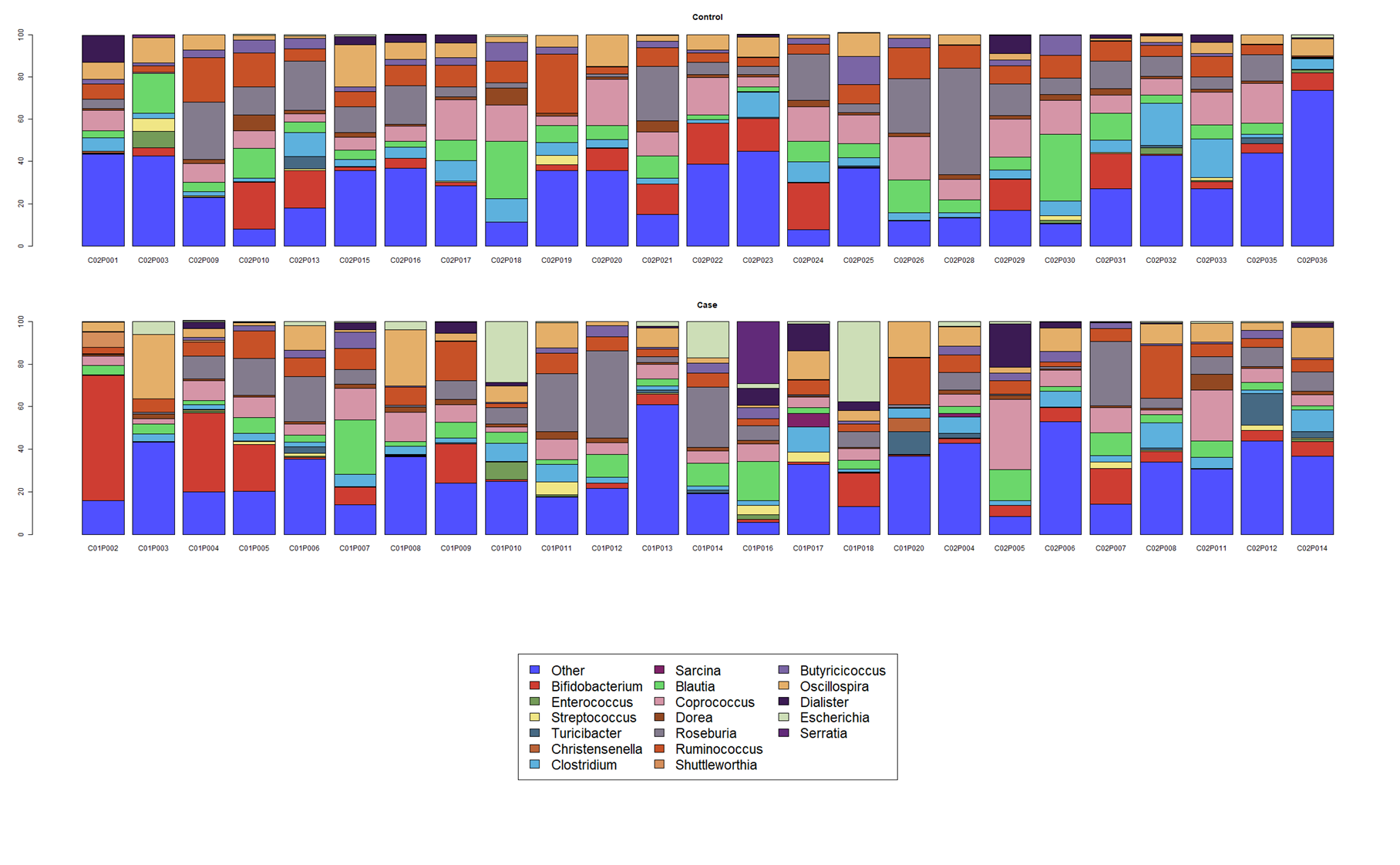

Supplement: Supplementary file 4 [file mmc4.zip › mmc4.png]
